# Contrast normalization contributes to a biologically-plausible model of receptive-field development in primary visual cortex (V1)

**DOI:** 10.1016/j.visres.2011.12.008

**Published:** 2012-02-01

**Authors:** Ben D.B. Willmore, Harry Bulstrode, David J. Tolhurst

**Affiliations:** a Department of Physiology, Anatomy & Genetics, University of Oxford, Sherrington Building, Parks Road, Oxford OX1 3PT, UK; b Department of Physiology, Development & Neuroscience, University of Cambridge, Downing Street, Cambridge CB2 3EG, UK

**Keywords:** BCM, Contrast normalization, Learning, Simple cells, V1, Coding, Sparse

## Abstract

Neuronal populations in the primary visual cortex (V1) of mammals exhibit contrast normalization. Neurons that respond strongly to simple visual stimuli – such as sinusoidal gratings – respond less well to the same stimuli when they are presented as part of a more complex stimulus which also excites other, neighboring neurons. This phenomenon is generally attributed to generalized patterns of inhibitory connections between nearby V1 neurons. The Bienenstock, Cooper and Munro (BCM) rule is a neural network learning rule that, when trained on natural images, produces model neurons which, individually, have many tuning properties in common with real V1 neurons. However, when viewed as a population, a BCM network is very different from V1 – each member of the BCM population tends to respond to the same dominant features of visual input, producing an incomplete, highly redundant code for visual information. Here, we demonstrate that, by adding contrast normalization into the BCM rule, we arrive at a neurally-plausible Hebbian learning rule that can learn an efficient sparse, overcomplete representation that is a better model for stimulus selectivity in V1. This suggests that one role of contrast normalization in V1 is to guide the neonatal development of receptive fields, so that neurons respond to different features of visual input.

## Highlights

► Contrast normalization can be introduced to a BCM neural network. ► The resulting network efficiently represents natural images. ► Contrast normalization prevents redundant representation of image structure. ► Neurally plausible model for neonatal development of receptive fields in V1.

## 1 Introduction

“Simple cells” are a group of neurons which perform approximately linear summation of patterns of light falling on the retina, whether simple stimuli such as gratings, lines and edges or more complex natural images (Hubel & Wiesel, 1959; Movshon, Thompson, & Tolhurst, 1978b; Smyth et al., 2003). Because such linear responses are mathematically tractable, simple cells have become the focus of numerous theoretical investigations into the principles that determine the structure of neural codes for sensory information. A number of such investigations have suggested that the population of simple cells forms a code that is optimized in some way for encoding stimuli which have the statistical structure found in natural images (Field, 1994; Hancock, Baddeley, & Smith, 1992).

One way to test this hypothesis is to develop a neural network learning rule that instantiates some current theory about optimal coding, and then train that network to represent natural stimuli. If the resulting network resembles the simple cell code, one has an “existence proof” which suggests that the theory may be one of the neural principles that underlie the structure of the simple-cell code. Numerous studies have taken this approach. Fyfe and Baddeley (1995) trained neural networks to perform Principal Components Analysis (PCA) on photographs of natural scenes. Although they found some similarities between the resulting networks and the receptive-fields of simple cells, the network failed to capture the fundamental fact that simple-cell receptive fields are localized in space. In contrast, a series of studies (Bell & Sejnowski, 1997; Caywood, Willmore, & Tolhurst, 2004; Olshausen & Field, 1996, 1997; van Hateren & van der Schaaf, 1998) have shown that neural networks which instantiate sparse, distributed learning rules (such as Independent Components Analysis or ICA) produce model neurons which share many of the basic tuning properties of real V1 simple cells. Of course, the anatomy of the LGN to V1 pathway ensures that receptive fields are likely to be localized since one part of V1 receives input about a small region of retina; but within that anatomical cause of localization, algorithms like ICA develop fields that are even more spatially localized.

These learning rules may, then, provide a theoretical account for the structure of simple-cell receptive fields. But what is the mechanism by which the receptive fields actually develop? The learning rules discussed above generally involve iterative refinement of model receptive fields. Now, the iterations may just be the algorithmic means by which a stable arithmetical solution is obtained such as in PCA; however, in the Olshausen-Field algorithm (Olshausen & Field, 1996), iteration is an explicit part of the learning mechanism. Perhaps, such iteration is actually a good model for the refinement of real neural receptive fields that is known to occur during the neonatal critical period, when an animal’s V1 receptive fields become matched to the visual features it sees during its early life (Blakemore & Cooper, 1970; Blakemore & Van Sluyters, 1975; Wiesel & Hubel, 1963).

However, these theoretical learning rules have not generally been implemented with neurally plausible mechanisms. For example, the Olshausen-Field rule requires a global error signal to be fed back through the network, and such a signal would require a veridical internal representation of the stimulus to compare with some reconstructed version. It is difficult to imagine how the veridical signal could arise and, indeed, reconstruction of the inputs may not actually be a goal of visual coding.

In contrast, one learning rule – the Bienenstock, Cooper and Munro (BCM, 1982) rule – does not require an error signal, and can generate plausible simple-cell receptive fields using only neurally-plausible Hebbian and anti-Hebbian learning rules at the level of individual synapses. Law and Cooper (1994) showed that the BCM rule, for a *single* neuron trained on natural images, produces a model receptive field which has many similarities to those of real simple cells. However, in its simplest form, the BCM rule provides no way for neurons to communicate with each other in order to form a meaningful coding *population*. We shall show that, without such communication, all the neurons in a BCM network will tend to represent similar features, forming a neural code that very redundantly represents just a small set of input features (the dominant ones in the input) but fails to represent much of the variability in the input. Such a neural code is unlike the real cortical code because, although individual model neurons do have sparse responses, coding is not well distributed across all members of the population (whose responses, therefore, will be highly correlated).

Now, it is well-established that neurons in primary visual cortex *do* communicate amongst one another. “Contrast normalization” is one result of this communication, where it is presumed that all neurons with similar receptive field locations mutually inhibit one another (Geisler & Albrecht, 1992; Heeger, 1992). A large variety of known nonlinearities in V1 neurons (e.g. response saturation, cross-orientation suppression, Albrecht & Hamilton, 1982; Bonds, 1989) can be accounted for in terms of the divisive arithmetic of contrast normalization (Heeger, 1992; Tolhurst & Heeger, 1997). It has been shown by Schwartz and Simoncelli (2001) that contrast normalization decorrelates the neural representation of visual information, and it is decorrelation that is needed to force a population of learning cells to take up different receptive-field shapes. Shouval, Intrator, and Cooper (1997) did introduce a “lateral” inhibitory term to the BCM model in order to show how ocular dominance and orientation columns might develop. Falconbridge, Stamps, and Badcock (2006) also have competition or lateral connectivity between the elements of their network, which also involves Hebbian learning. However, the implications of contrast normalization for biologically-plausible learning rules and for the usefulness of the resultant coding bases have not yet been considered, even though decorrelation is clearly seen as one goal of an efficient visual code (Schwartz & Simoncelli, 2001). We will model contrast normalization as posited by Heeger (1992) to explain many non-linear behaviors of visual cortex neurons.

In the present study, we have investigated whether combining the well-established mechanism of contrast normalization with the BCM rule can produce a model neural population in which all neurons contribute equally to coding of visual information, and in which redundant representation of visual information is reduced. We have added a contrast-normalization term to the BCM rule, and have measured various aspects (Willmore & Tolhurst, 2001) of the visual code in the resulting neural population to demonstrate how the simple-cell code might arise. Blais et al. (1998) did examine the statistics of the responses of single trained neurons to natural images; this allows understanding of, say, that which we have called “lifetime sparseness” but examining single neurons cannot address whether the population code has proper coverage, for instance.

## 2 Methods

### 2.1 Image acquisition

We used a carefully calibrated and linearized set of 64 256 × 256-pixel black-and-white photographs of landscapes (16 photographs), plants and trees (16), animals and people (16) and man-made objects (16). The acquisition and calibration procedure is described in detail by Tolhurst, Tadmor, and Tang (1992). Briefly, photographs were taken using Kodak Tmax-400 35 mm low gamma film, at known shutter speeds. Each roll of film included photographs of a chart of 15 Munsell grey-papers whose luminances were measured with a spot photometer (Minolta Inc.), and varied over a 20× range. By taking photographs of these charts at a range of shutter speeds and with a 10× neutral-density filter, the low gamma negatives could be converted into luminance values spanning more than 3 log units.

### 2.2 Model of retinal processing

First, the logarithm was taken of all pixel values. The log-transform is an accepted model of light adaptation in individual photoreceptors (Field, 1994), although it does not model any retinal “network adaptation”. Then, to model spatial summation and lateral inhibition in retinal ganglion cells (RGCs), we convolved each 256 × 256 pixel photograph with a difference-of-Gaussian (DoG) filter, using parameters typically found in retina or LGN of various species (Benardete & Kaplan, 1997; Irvin, Casagrande, & Norton, 1993; Linsenmeier et al., 1982). The standard deviation of the central (excitatory) and surround (inhibitory) Gaussians were 0.75 and 2.25 pixels, and their volumes were balanced to give a zero-D.C. filter. This ratio is typical of real RGCs or LGN cells, but it produces a filter that is not much like the image-whitening filter used by, e.g. Olshausen and Field (1996). The low and high spatial frequency fall-offs of RGCs are relatively gradual; they do not have the correct low frequency slope to counter the supposed 1/*f* amplitude spectrum of natural images, and they do not have the very rapid high spatial frequency fall off of the whitening filter. Real RGCs may filter out low spatial frequencies but they do not seem to whiten or greatly exaggerate high frequencies. To avoid edge effects, the edges (10 pixels) of each convolved image were discarded. Finally, the 236 × 236-pixel convolutions were cut into many randomly-selected 16 × 16-pixel patches, each representing the response of 16 by 16 RGCs to a small part of image. Each patch was standardized by subtracting its mean value and dividing by its standard deviation. It should be noted that all DoG fields had the same center diameter, even though it is known that P- and M-cells differ in their center diameters (Croner & Kaplan, 1995; Derrington & Lennie, 1984).

### 2.3 BCM learning rule

We modeled an array of 256 cortical neurons, each receiving inputs from a common 16 × 16 array of RGCs; we imagine that the LGN acts simply as a relay, and that the two populations of ON and OFF center input cells can be represented in the sign of the model RGC responses (which can be positive and negative). The responses of the RGCs to each stimulus are described by *d_i_*. The weight matrix (*m_ij_*) describes the strengths of the synapses between each of the *i* RGCs and the *j* cortical neurons. The weight matrix is initially set to random values between −1 and 1. Then randomly cropped image stimuli are presented in turn for several million iterations. Every time a stimulus is presented, the activations, *c_j_*, of all cortical neurons are calculated as follows:

(1) $$c_{j}=s\left(\underset{i}{\sum }m_{\mathrm{ij}}d_{i}\right)$$

where the sigmoidal (thresholding) output nonlinearity (Bienenstock, Cooper, & Munro, 1982), *s*, is given by:

(2) $$s(r)=\left\{\begin{array}{cc} k_{1}\tan h(r) & \text{for}\;r>0\\ k_{2}\tan h(r) & \text{for}\;r<0 \end{array}\right)$$

where *r* is the linear activation. We used *k*_1_ = 25 and *k*_2_ = 1, following Cooper et al. (2004).

The nonlinear activation of each neuron is subject to a learning threshold, *θ_j_*. After each stimulus presentation, the weight matrix (*m_ij_*) is updated according to the product of the difference between the activation of each neuron and its present learning threshold on the one hand and the activity, *d_i_*, of the corresponding RGC on the other:

(3) $$\delta m_{\mathrm{ij}}=\eta c_{j}(c_{j}-\theta_{j})d_{i}$$

where *η* determines the learning rate (initially, *η* = 10**^−5^**). This results in enhancement of weights when the neuron’s activation is suprathreshold (Hebbian learning), but reduction of weights when the activation is subthreshold (anti-Hebbian). The learning threshold for each neuron, *θ_j_*, is also altered after each stimulus presentation:

(4) $$\delta \theta_{j}=\left(c_{j}^{2}-\theta_{j}\right)/\tau$$

where *τ* determines the rate of change of *θ_j_*, and is set to 1000. *η* is also decreased by 0.1% once every 1000 iterations. These changes in learning threshold and learning rate lead to a stable solution (generally within a million iterations, or 1000 changes of *η* in Eq. (3)). It is clear, so far, that the learning in any one of the *j* neurons is unaffected by the responses or the learning of any of the other 255 neurons.

### 2.4 Normalized BCM learning rule

The normalized BCM (NBCM) rule is identical to the standard BCM, except that divisive normalization is applied after the activation calculation (Eq. (1)). The activation *c_j_* of each neuron is divided by a normalizing signal which is derived from the sum of the squared activations of all neurons in the network to that image patch (Heeger, 1992):

(5) $$c_{j}^{\prime }=\frac{\beta c_{j}}{\alpha +\sqrt{\underset{i}{\sum }c_{i}^{2}}}$$

**Fig. 1:**
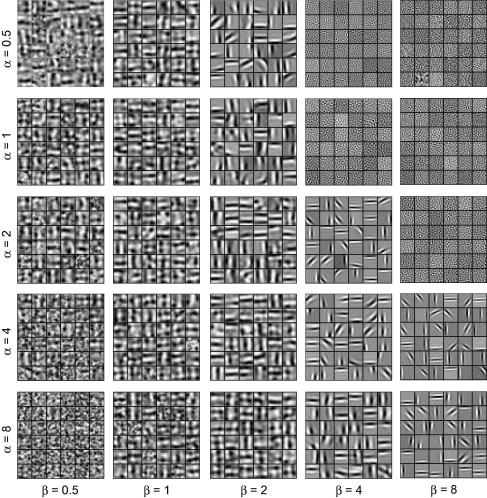
Effect of contrast normalization parameters on receptive field structure. When *α* ≫ *β* (bottom left), NBCM produces full-field low-frequency non-oriented receptive fields. When *β* ≫ *α* (top right), it produces white noise receptive fields. When *α* and *β* are comparable, NBCM produces oriented, localized receptive fields which resemble the receptive fields of V1 simple cells; the optimal spatial frequencies are affected by the exact values of *α* and *β*.

*α* resembles the semi-saturation constant in the contrast/response formulations of Albrecht and Hamilton (1982) and Heeger (1992), and our Eq. (5) is very similar to Albrecht and Hamilton’s Naka–Rushton equation and to Heeger’s simple formulation of contrast normalization in visual cortex. The parameters *α* and *β* determine the strength of normalization relative to the responses *c_j_*. We investigated the effects of these parameters on the resulting receptive fields (see Fig. 1 ). We found that, when *α* ≫ *β*, NBCM produced full-field low-frequency non-oriented receptive fields, and, when *α* ≪ *β*, it produced white noise patterns. However, when *α* and *β* were comparable, the receptive fields were localized and oriented, resembling the receptive fields of V1 simple cells. For comparable *α* and *β*, the receptive fields always had similar shape, but changes in *α* and *β* did then cause variations in the fields’ spatial frequency preferences (e.g. in Fig. 1, compare *α* = 1, *β* = 2 with *α* = 2, *β* = 4). The values of *α* and *β* (in Eq. (5)) and *k*_1_ and *k*_2_ (in Eq. (2)) were all pragmatically found to match the values of *r* (Eq. (2)) typically found with our combinations of image data and field weights. We discuss two parameter pairings in Section 3: *α* = 1, *β* = 2 and *α* = 2, *β* = 4.

### 2.5 Measures of coding quality

To assess the codes formed by the BCM and NBCM receptive fields, we used several measures (Vinje & Gallant, 2000; Willmore & Tolhurst, 2001).

#### 2.5.1 Reconstruction of receptive fields

The BCM learning algorithm produced sets of RGC input *weights* to the cortical neurons. However, for the analysis of population coding statistics, it was first necessary to use those weights to reconstruct the receptive fields, i.e. to determine how the neurons would respond to visual stimuli (e.g. the image fragments after the log transform, but before filtering by the RGCs) without the intermediate step of calculating the responses of the array of RGCs. For each model neuron, the 16 × 16 array of RGC weights was embedded in a 64 × 64 2D array of zeroes. The 2D Fourier transform was taken and multiplied by the Fourier transform of the same DoG used for image pre-filtering (Section 2.2) to give the receptive field. Each field was then cropped back to 16 × 16 pixels, and standardized by subtracting its mean and dividing by its standard deviation.

#### 2.5.2 Lifetime sparseness

To measure the lifetime sparseness of the receptive fields, we first calculated their responses to a set of 1000 log-transformed image fragments, taken from the same parent images which had been used to train the receptive fields, though these had been convolved with the RGC DoG for training. We then calculated the sparseness of each receptive field’s responses to the many image fragments, using the Vinje and Gallant sparseness measure (Vinje & Gallant, 2000):

(6) $$S=1-E{\left[r\right]}^{2}/E[r^{2}]$$

where *E*[*x*] denotes the expected value of *x.* To obtain a single value for the whole set of receptive fields, the mean of all the lifetime sparseness values was used.

#### 2.5.3 Population sparseness

Lifetime sparseness is relatively easy to measure for single real neurons (e.g. Tolhurst, Smyth, & Thompson, 2009) but it is a poor measure of how populations of neurons encode images (Willmore & Tolhurst, 2001). Population sparseness is surprisingly poorly correlated with lifetime sparseness for some coding schemes. To measure population sparseness, we used the responses and the same equation as for lifetime sparseness. However, the sparseness calculation was applied to the set of responses of all 256 receptive fields to a single image separately, and the resulting values were then averaged across all 1000 image patches.

#### 2.5.4 Dispersal

Dispersal is a measure of how much each receptive field contributes to the representation of the variance in the whole set of images (Willmore, Watters, & Tolhurst, 2000); for instance, a PCA code would be poorly dispersed because most of the image variance would be coded by a few fields. To measure dispersal, we used the same set of responses as for the sparseness calculations. We calculated the standard deviation, *σ_j_*, of each receptive field’s lifetime responses. The dispersal, *D*, is the normalized sum of the standard deviations:

(7) $$D=\underset{j}{\sum }(\sigma_{j}/\max(\sigma_{j}))$$

#### 2.5.5 Orthogonality and rank

We determined the overall *orthogonality* of the array of fields (not RGC weights) by calculating the dot products between each field and every other field, averaging those values, and subtracting the average from 1.0. A perfectly orthogonal set would give an orthogonality of unity. We also found the *rank* of the 2D matrix whose rows are the flattened receptive fields, using the Matlab (The Mathworks Inc.) *rank*() function with tolerance of 2.5.

#### 2.5.6 Coverage (reconstruction of sinusoids)

We created a complete set of 256 sinusoids on a 16 × 16 grid, each with vector length 1. We used linear encoding to represent each sinusoid using the learned cortical receptive fields, and then decoded each sinusoid using the inverses of the cortical fields and measured the mean square error, *m_i_*, of the decoding. We then defined the reconstruction quality to be:

(8) $$R=1-E[m_{i}]$$

Thus, perfect reconstruction (coverage) would give *R* = 1. The inverse fields were estimated from the fields using the Matlab function *pinv*(), again with tolerance of 2.5. As well as finding a single summary value for coverage, this allows us to determine which parts of the 2D spectrum are over- or under-sampled by the coding set.

### 2.6 Measurements of receptive field parameters

Before taking the inverse Fourier transform of the receptive fields (above), we calculated various parameters of the 64 × 64 2D Fourier amplitude spectrum of each receptive field. Preferred spatial frequency and orientation were defined as the spatial frequency and orientation of the single strongest coefficient in the amplitude spectrum. Spatial frequency bandwidth was defined conventionally as the distance in octaves between the extreme half-maximum points of the spectrum. Orientation bandwidth was defined conventionally as the difference in degrees between the extreme half-maximum points of the spectrum.

## 3 Results

We sought to examine the effect of combining contrast normalization and the BCM rule in the training of a network of neurons representing natural images. To do this, we trained two networks to represent the same set of images under identical conditions. One network used the standard BCM rule (where each of 256 neurons learned independently) and the other used our normalized BCM (NBCM) rule (where the neurons interacted through inhibitory connections in the way suggested by Heeger (1992)).

### 3.1 Qualitative effects of contrast normalization

**Fig. 2:**
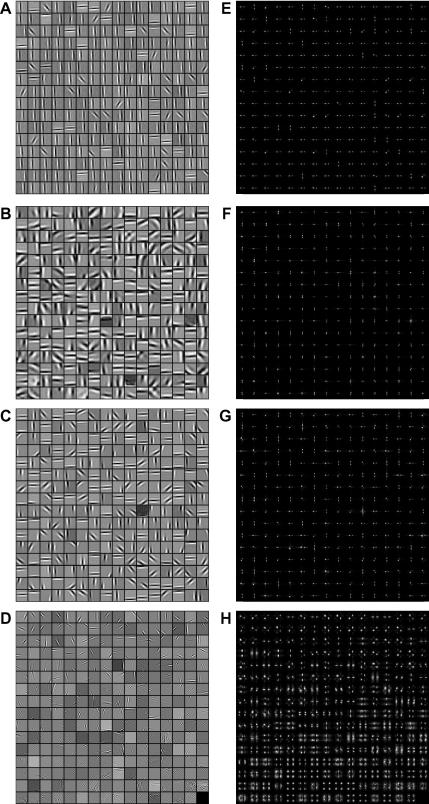
Receptive fields generated by the standard and contrast-normalized BCM algorithms, with Independent Components Analysis (ICA) for comparison. (A) The standard BCM algorithm, when applied to natural images, generates receptive fields containing patterns of parallel light and dark bars. Each receptive field is reminiscent of the tuning found in the simple cells of mammalian V1. However, when viewed as a population, many of the BCM receptive fields are very similar to one another, so that the same visual information (primarily horizontal and vertical structure) is represented redundantly by many receptive fields. (B) The NBCM algorithm is similar to the standard BCM, but incorporates contrast normalization. This results in a wider variety of receptive fields, and much lower redundancy (here, *α* = 1 and *β* = 2). (C) With a different choice of parameters (*α* = 2, *β* = 4), the fields have high spatial frequencies, but similar structure. (D) Receptive fields produced by ICA of the same images cover a range of positions and orientations, but are of generally high spatial frequency. (E–H) Fourier power spectra of the receptive fields in (A–D). The uniformity of the standard BCM receptive fields (D) is particularly noticeable in their power spectra.

Fig. 2 provides a qualitative understanding of the differences between the receptive fields produced by the two networks. Fig. 2A shows the receptive fields produced by the standard BCM rule; each small grey box represents a single receptive field. The receptive fields are shown as elongated patterns of light and dark bars representing ON and OFF regions, which bear a superficial resemblance to the receptive fields of V1 simple cells (DeAngelis, Ohzawa, & Freeman, 1993; Hubel & Wiesel, 1959; Jones & Palmer, 1987). However, the BCM receptive fields have two striking properties. First, the receptive fields are very elongated, in most cases stretching along most of the extent available to them. Secondly, when seen as a population, it is clear that many of the receptive fields are very similar to one another. Rather than forming a heterogeneous population with a range of tuning parameters, most of the BCM receptive fields are aligned along the *y*-axis, with a few receptive fields aligned to the *x*-axis or at 45°. Fig. 2E shows the Fourier amplitude spectra of the receptive fields, and shows that the spatial frequency and orientation preferences of the receptive fields are indeed very similar to one another.

Fig. 2B shows the receptive fields produced by the NBCM network, using parameters *α* = 1, *β* = 2 (Eq. (5)). Like the standard BCM, these receptive fields are elongated patterns of light and dark bars that are superficially similar to V1 simple cells. However, they differ from the standard BCM receptive fields (Fig. 2A) in several important ways. First, many of them have lower spatial frequency preferences (the parallel bright and dark stripes are broader); this is particularly clear in Fig. 2F, which shows the Fourier amplitude spectra of the receptive fields. Second, they are relatively less elongated than the standard BCM receptive fields (since the spatial frequency preferences are lower, but the length of the receptive fields is similar). Finally, and most significantly, the NBCM receptive fields seem to form a much more heterogeneous population than the standard BCM receptive fields, particularly because they occur with a greater range of preferred orientations.

Fig. 2C and G shows the results of training the NBCM network with a different choice of *α* and *β* values (*α* = 2, *β* = 4). Again, The NBCM model has produced fields similar to those in V1, and significantly with a range of orientation optima. The change in model parameters has, however, resulted in a shift of the spatial frequency tuning towards higher frequencies (the parallel bright and dark stripes are slightly narrower in Fig. 2C than B).

For comparison, Fig. 2D and H shows the results of running Independent Components Analysis (ICA) on the same image set (Bell & Sejnowski, 1997; van Hateren & van der Schaaf, 1998). Approximately the first half of the fields are localized in orientation and spatial frequency, but later fields (which account for decreasing proportions of variance in the image set) have very high spatial frequencies and are not well-localized in orientation.

### 3.2 Effect of contrast normalization on spectral coverage

**Fig. 3:**
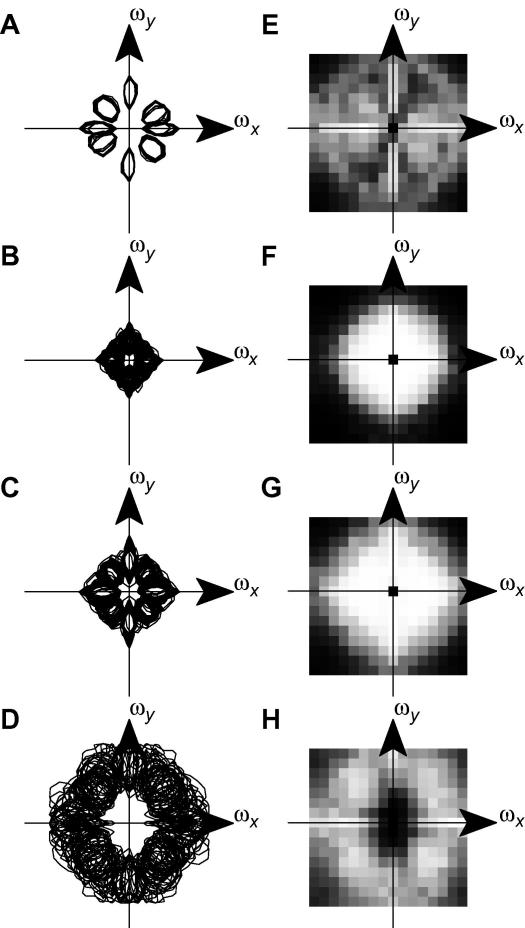
Frequency tiling of receptive fields generated by the BCM, NBCM and ICA algorithms. (A) Fourier-space plot showing the half-maximum contour for each of the BCM receptive fields shown in Fig. 2A. Although 256 receptive fields are represented here, they have very similar Fourier spectra, and so only four different spectral profiles are discriminable. Also, there are noticeable gaps in the orientation coverage of these profiles. (B and C) Fourier-space plots showing the half-maximum contour for the NBCM receptive fields shown in Fig. 2B and C, respectively. Unlike the BCM receptive fields, there is a wide variety of NBCM receptive fields, and, at least for low spatial frequencies, all orientations are well sampled. (D) Receptive fields produced by ICA. Only the first 128 fields are shown, because later fields do not have Gabor-like structure. The ICs cover a range of orientations, though there is a gap at low spatial frequencies. (E) Ability of the standard BCM receptive fields to support reconstruction of sinusoids. Although the reconstruction of horizontal and vertical orientations is reasonable, there are gaps in the coverage of all other orientations. (F and G) In contrast, similar maps for the NBCM (corresponding to B and C, respectively) show that all orientations can be reconstructed accurately. The only gaps in coverage are at high spatial frequencies which were not present in the input images (due to DoG filtering). (H) A similar map for ICA confirms that there is a gap in coverage at low spatial frequencies.

To form a complete, general code for visual information, a set of receptive fields must represent *all* frequencies and orientations of visual information. Inspection of Fig. 2A and E suggests that a population of receptive fields produced by the standard BCM model may not meet this criterion. To investigate whether this is in fact the case, we first inspected the Fourier amplitude spectra of the receptive fields. Fig. 3 A shows the half-maximum contours of the spectra (from Fig. 2E) of all the standard BCM receptive fields (Fig. 2A), plotted on one set of axes. Although there are 256 receptive fields in total, there are only four distinct contours (each of which has two symmetric lobes), corresponding approximately to 0°, 45°, 90° and 135°. This confirms that the limited variety of receptive fields produced by the BCM model does not cover all possible frequencies and orientations.

In contrast, similar contour plots for the NBCM models (Fig. 3B and C) show that the NBCM receptive fields cover orientation space much more evenly, and there are no significant gaps in the orientation coverage. The NBCM fields seem to cover lower spatial frequencies than the BCM fields, but this depends on the choice of NBCM parameters.

Fig. 3D shows a similar plot for the ICA receptive fields (only the well-localized first half of the fields are shown). Like the NBCM fields, ICA shows good orientation coverage. Unlike the NBCM, however, the ICA fields have very high spatial frequencies and there is a gap at low spatial frequency (see also Willmore & Tolhurst, 2001).

To determine whether these differences in orientation and frequency coverage actually affect the usefulness of the code, we tested the ability of each set of receptive fields to reconstruct a set of test stimuli, which comprised an odd–even pair of sinusoids at every possible orientation and spatial frequency in the 16 × 16-pixel grid. We encoded each sinusoid using the standard BCM receptive fields, and then decoded it again, using linear encoding for each step (see Section 2). We then measured the RMS encoding error for each reconstructed sinusoid. The resulting errors are shown in Fig. 3E–H, arranged according to the spatial frequency and orientation of the sinusoids. White indicates perfect reconstruction, and black indicates a large reconstruction error. This representation provides a measure of the ability of the code to represent every possible spatial frequency and orientation. It reveals several failures of the standard BCM code (Fig. 3E). First, the highest frequencies (corners of the plot) are very poorly represented. This is because the DoG-filtering applied to the input images removes most power at these frequencies, and so the neurons were not in fact trained to represent them. However, there is also relatively poor coverage of the lowest spatial frequencies (the center of the reconstruction spectrum). More importantly, only the few sinusoids that are aligned on the *x-* and *y-*axes show near-perfect reconstruction. All other orientations are relatively poorly represented by the standard BCM receptive fields. This is consistent with the disjoint orientation coverage observed in Fig. 3A.

To determine whether contrast normalization improved the representational capacity of the network, we performed the same analysis for the NBCM receptive fields. The reconstruction errors are shown in Fig. 3F and G, on the same grey-level quality scale as Fig. 3D. The reconstruction errors are substantially lower for most sinusoids, and in particular, the reconstruction of non-axially-aligned sinusoids is greatly improved. As expected, there are gaps at the high spatial frequencies that were removed by DoG filtering, but apart from those, all frequencies are well-represented by both sets of NBCM receptive fields, even though these had rather different optimal spatial frequencies. For comparison, Fig. 3H shows a similar analysis for the first half of the ICA fields. This confirms the gap in coverage of low spatial frequencies.

To quantify the overall representational capacity of the two networks, we measured the reconstruction errors averaged over all sinusoids (see Table 1 ); the standard BCM had an average error of 0.61 whilst the NBCM had an average error of 0.44 (Fig. 3F) or 0.31 (Fig. 3G). This difference suggests that contrast normalization substantially increases the representational capacity of the network, making it comparable with ICA (average error 0.28).

### 3.3 Effect of contrast normalization on redundancy

**Fig. 4:**
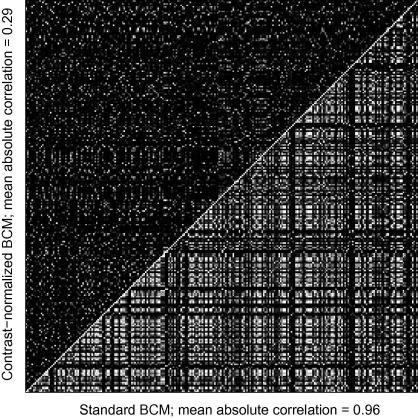
Orthogonality of receptive fields produced by the standard BCM and NBCM models. The lower right triangle shows the absolute scalar product between each pair of receptive fields produced by the standard BCM model. The values are normalized so that the scalar product of one field by itself would give a value of 1. Larger absolute scalar products (i.e. less orthogonality) are indicated by brighter pixels. The upper left triangle shows a similar plot for the NBCM model (*α* = 2, *β* = 4). To estimate the overall orthogonality of the receptive fields, we take the mean of these values and subtract that mean from 1, to give a measure that increases to 1 for perfectly orthogonal fields. The resulting orthogonality is lower for the standard BCM model (0.24) than the NBCM model (0.073), indicating that the BCM receptive fields form a relatively redundant code for visual information.

The standard BCM model contains many very similar receptive fields. We have already shown one disadvantage of this: that the network fails to represent some visual information. A second disadvantage is that this repetition results in a highly redundant code. To quantify the effect of contrast normalization on the redundancy of the receptive fields, we measured the absolute value of the scalar products between all pairs of receptive fields within each network (i.e. their relative orthogonality). This is a simple way to estimate the redundancy of the code because it measures the similarity of the receptive fields, and therefore how much redundant information they transmit according to a pairwise correlation model. The resulting scalar product magnitudes (black represents zero, bright represents values up to unity) are shown in Fig. 4 for the BCM and for one of the NBCM models. The bottom-right triangle shows values for the standard BCM receptive fields; the top-left triangle shows values for the NBCM receptive fields of Fig. 2C. The brightness scale is the same in each case. The values are generally higher for the standard BCM (mean = 0.24) than for the NBCM (mean = 0.073). This indicates that the NBCM contains receptive fields that are less similar to one another on average, and are less likely to transmit redundant information; i.e. that contrast normalization reduces the redundancy of the network. The measure of *orthogonality* in Table 1 is given as one minus these averages.

### 3.4 Effect of contrast normalization on other coding properties

To produce a more complete description of the effects of contrast normalization on the BCM network, we measured other coding properties proposed by Willmore, Watters, and Tolhurst (2000) and Willmore and Tolhurst (2001). The results are shown in Table 1.

First, we measured the *rank* of the receptive field matrix; this provides a measure of how completely the code represents visual space, similar to the reconstruction error measured above. We find that the rank of the standard BCM receptive fields is only 99 (out of a possible 256). The NBCM (*α* = 2, *β* = 4) has substantially higher rank (144), indicating that it provides better coverage of visual space; and the NBCM with *α* = 1, *β* = 2 had an even higher rank of 175, comparable to ICA (168).

We also measured the *lifetime* *sparseness* and the *population* *sparseness* (Willmore & Tolhurst, 2001) of the standard BCM and NBCM; we find that the NBCM has slightly higher population sparseness at the expense of slightly lower lifetime sparseness. We find that the *dispersal* (also known as *distributedness*) of the standard BCM code is higher than that for the NBCM code. Although high dispersal might be a requirement of a good “sparse, distributed” code (Field, 1994), the higher dispersal of the standard BCM actually reflects the code’s higher redundancy or lower orthogonality.

### 3.5 Effect of contrast normalization on orientation and spatial frequency tuning

The above analyses suggest that the NBCM rule produces more desirable coding properties than the standard BCM. However, the key reason to be interested in the BCM rule is that, when trained on natural images, it produces receptive fields that, at first sight, resemble those of V1 simple cells. To determine whether the NBCM rule also has this desirable feature and whether the better coding properties also reflect better matches to real V1 simple cells, we measured various parameters of the standard BCM and NBCM receptive fields, and, where possible, compared these with the parameters of real V1 neurons measured in previous studies (De Valois, Albrecht, & Thorell, 1982; De Valois, Yund, & Hepler, 1982; Movshon, Thompson, & Tolhurst, 1978a; Tolhurst & Thompson, 1981).

**Fig. 5:**
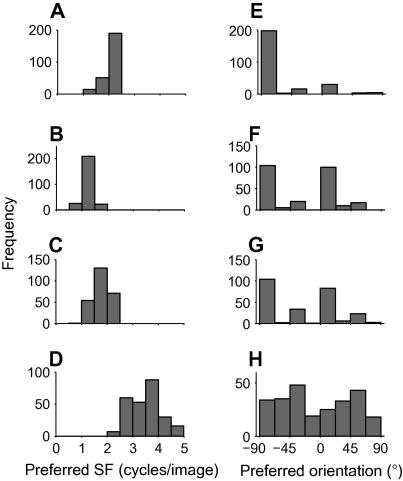
Comparison of the spatial frequency and orientation tuning of receptive fields produced by the standard BCM, NBCM and ICA models. (A–D) The preferred spatial frequencies of the BCM (A) and NBCM (B and C) receptive fields are lower than those of ICA (D). (E–H) The standard BCM model (E) produces receptive fields which all have similar orientations, and these are dominated by −90° (vertical). The NBCM (F and G) and ICA (H) models produce a much wider variety of orientations, though some quantization is still visible in the NBCM fields.

Fig. 5 A–D and E–H, respectively show the preferred spatial frequencies and orientations of the receptive fields of Fig. 2. The NBCM receptive fields (5B–5C) have similar preferred frequencies (perhaps slightly lower) to the standard BCM (5A); the exact values depend on the parameters used for the NBCM. ICA has higher preferred spatial frequencies (5D). The preferred orientations of the standard BCM model (5E) cluster around −90° (vertical), and other orientations are barely represented. The NBCM (Fig. 5F and G) produces a more even distribution of preferred orientations, though there are still prominent peaks at around −90° and 0°. ICA produces a more even distribution across orientation (5H) though this may be partly because many of the ICA fields did not have clear orientation tuning.

**Fig. 6:**
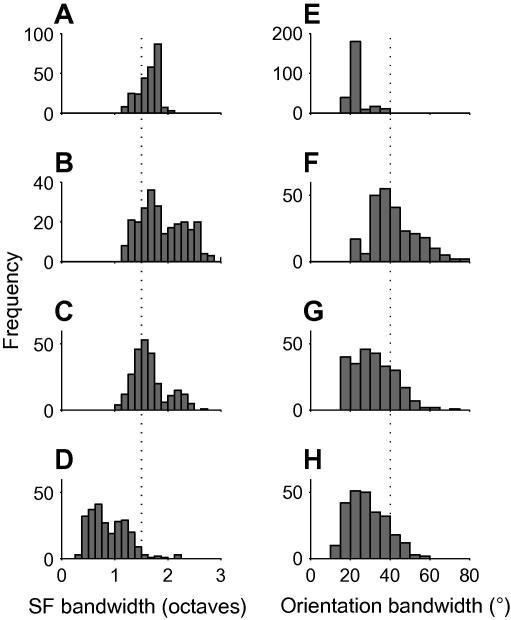
(A–D) Spatial frequency bandwidth of the BCM, NBCM and ICA receptive fields. The BCM (A) and NBCM (B and C) algorithms produce receptive fields with similar spatial frequency bandwidths, and these are a good match for the bandwidths measured for cat and macaque V1 neurons (De Valois et al., 1982a; Tolhurst and Thompson, 1981), which average 1.5 octaves (marked by dotted line). ICA (D) produces receptive fields with somewhat lower spatial frequency bandwidth. (E–H) The standard BCM algorithm (E) produces receptive fields which are very long and thin, and this is reflected in their low orientation bandwidths. The NBCM and ICA algorithms (G and H) produce less extended receptive fields, and as a result, their orientation bandwidths are a better match for the bandwidths measured for macaque V1 by (De Valois et al., 1982b), which averaged 40° (dotted line).

Fig. 6 A–D and E–H, respectively show the spatial frequency and orientation bandwidths of the receptive fields. The spatial frequency bandwidths of the BCM (6A) and NBCM (6B and 6C) are very similar, peaking around 1.5 octaves; however, some of the higher spatial frequency NBCM (6B) set have broader bandwidths. This figure of 1.5 octaves is in good agreement with the peak for cat and macaque V1 neurons (De Valois, Albrecht, & Thorell, 1982; Movshon, Thompson, & Tolhurst, 1978a; Tolhurst & Thompson, 1981), indicated by the dotted line. In contrast, the ICA fields (Fig. 6D) have substantially narrower SF bandwidths.

The orientation bandwidths of the standard BCM receptive fields (Fig. 6E) are low, with a peak around 25°. This reflects their extreme elongation. The orientation bandwidths of the NBCM receptive fields (6F and 6G) are closer to 40°, the peak for macaque V1 neurons (De Valois, Yund, & Hepler, 1982), indicated by the dotted line. ICA fields generally have lower orientation bandwidth (6H). Altogether, the spatial frequency and orientation bandwidths suggest that the NBCM receptive fields are more similar to V1 neurons than the standard BCM or ICA receptive fields.

### 3.6 Localization of receptive fields

**Fig. 7:**
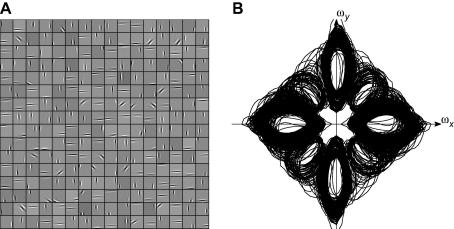
(A) 32 × 32 pixel receptive fields produced by the NBCM model (only 256 of 1024 are shown), showing the spatial localization of the fields. (B) Fourier-space plot showing the half-maximum contour for each of the NBCM receptive fields shown in A. The spectra of the 32 × 32 fields are qualitatively similar to those of 16 × 16 fields (see Fig. 3B and C).

An important feature of V1 receptive fields is that they are spatially localized. The 16 × 16 pixel receptive fields used in this paper appear, in many cases, to be similarly localized, but it is hard to be sure because many of them are clipped by the edges of the pixel grid. To confirm that they are truly localized, we trained the NBCM on the same images as before, but using a 32 × 32 pixel grid. The resulting fields are shown in Fig. 7 A. Although some fields are still clipped, it is clear that many of them are genuinely localized within the 32 by 32 grid. The half-maximum contours (Fig. 7B) of these fields are qualitatively similar to those shown in Fig. 3B,C, and the receptive field parameters are similar to those plotted in Figs. 5B and C and 6B and C.

The localization of the fields of real V1 neurons will be first determined by the retinotopic mapping of LGN afferents to V1. Any V1 neuron can receive direct activation from LGN neurons subserving only a localized regions of space. Our simulations (especially with the 32 by 32 grid) show that the NBCM (and the BCM) produce fields localized even within the anatomical sampling. PCA (Hancock, Baddeley, & Smith, 1992) would produce unrealistically-shaped “fields” that filled whatever grid we chose, with no localization within that grid.

## 4 Discussion

The aim of this study was to create a neural network that can learn to form a sparse, dispersed representation of natural images without requiring an error signal to be fed back through the network. To do this, we combined the BCM rule – which employs neurally plausible Hebbian synaptic learning – and contrast normalization – a mechanism which is believed to exist in primary visual cortex (Heeger, 1992). We find that the combination of these two neurally plausible mechanisms (which we call the NBCM rule) produces a population of receptive fields with several interesting advantages over those produced by the standard BCM rule. Now, the standard BCM rule does produce receptive fields which are orientation and spatial-frequency tuned, and are superficially similar to the receptive fields of real V1 neurons (Law & Cooper, 1994). However, they do not behave as a meaningful population; instead, the majority of receptive fields are either axially-aligned (with a few oblique), leaving gaps in the orientation coverage of the population code. Also, the receptive fields are much more elongated than those of typical V1 neurons. The axial alignment follows the usual bias in our natural image fragments towards vertical and horizontal elements.

**Fig. 8:**
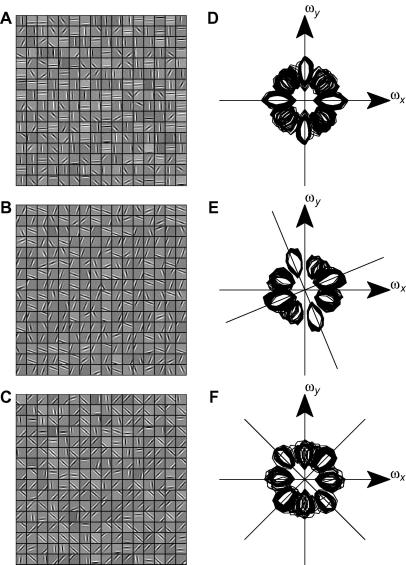
The NBCM receptive fields depend on the statistical structure of underlying images; they are not artifacts produced by quirks of the pixel grid. (A) Receptive fields produced from circularly-windowed images are qualitatively similar to those from square images (Fig. 3B and C). (B and C) When the images are rotated by 22.5 (B) or 45° (C), the receptive fields rotate accordingly. (D–F) Fourier-space plots showing the half-maximum contours for the NBCM receptive fields shown in A–C. The effect of rotation (E and F) is particularly clear in this representation, matching the rotation of the images (oblique axes).

The NBCM receptive fields form a more heterogeneous set (Fig. 2B and C) with a wider range of orientation preferences (Figs. 3B and C and 5F and G), although there are still biases towards verticals and horizontals. They form a more complete code (Fig. 3F,G), with less redundancy (Fig. 4). Finally, the orientation tuning bandwidths of the NBCM receptive fields are more similar to those of real V1 neurons (Fig. 6). The remaining biases in receptive field orientation to verticals and horizontals result from biases in the vertical and horizontal feature content of our sample of natural images. The bias does not arise from our use of a square sampling and training grid. Fig. 8 shows that, when we rotated the images before cutting them into 16 by 16 pixel squares, the NBCM receptive fields rotated to follow the image features and were not now parallel to the sampling grid. Fig. 8A and D shows the effect of applying a circular window to the image fragments; Fig. 8B and E shows the effect of a 22.5° image rotation; Fig. 8C and F shows a 45° rotation.

We have compared the NBCM fields with those generated by ICA (Bell & Sejnowski, 1997; van Hateren & van der Schaaf, 1998) and, in general, we find that the NBCM fields are a better match to the tuning properties of real visual cortex neurons and also that they comprise a more useful code with better coverage.

### 4.1 Why does contrast normalization have these effects?

The standard BCM rule has no mechanism for communication between neurons. Every neuron is connected to every input, but there are no lateral connections. Thus, there is no active mechanism that prevents neurons from redundantly representing the same visual information. As a result, all neurons tend to learn to respond to the dominant energy in the stimulus set, ignoring weaker components. In practice, different BCM neurons do not have identical receptive fields, but this is only because different neurons are initialized with different sets of random weights, and so they develop slightly different receptive fields reflecting local minima in the learning process. Without communication between neurons, a population of BCM neurons will learn to respond to a few dominant regularities in the images, producing a redundant code. In our set of 64 photographs, vertical features are dominant.

Contrast normalization is a neurally plausible form of lateral communication between neurons (Carandini & Heeger, 1994; Grossberg, 1988; Heeger, 1992; Marr, 1982). It modulates the response of each neuron according to the overall activity level of the population. When only a few neurons respond to a given input image, the contrast normalization signal is small, and so those neurons which are responding can produce strong responses. However, if a large number of neurons respond strongly to a given input, the contrast normalization signal is large, and all responses are consequently reduced. If, by chance, a large number of neurons respond to a given image, contrast normalization reduces all of their responses. Then, according to the BCM rule, this in turn reduces the amount of synaptic modification that each neuron undergoes in response to that image. Thus, image features which are already well represented by the population tend to produce less synaptic modification than those which are poorly represented by the population. Over many iterations, this decreases the chances that two neurons will respond to the same stimuli, and increases the chance that less dominant features in the input will be represented by the population. The result is a heterogeneous code in which different neurons learn to respond to different energy in the stimulus.

### 4.2 Comparison with physiological data

The NBCM rule produces model neurons which constitute a better model of the responses of real cortical neurons than the standard BCM rule. Like the real neural code, the NBCM code comprises Gabor-like filters at a range of orientations, and the tuning of the filters resembles the tuning of V1 neurons. However, the model and real codes are still different in two respects. First, V1 neurons have a wide range of spatial frequency preferences, whereas the NBCM neurons all have very similar frequency tuning. A similar problem has been observed with previous neural network models of V1 (Bell & Sejnowski, 1997; Olshausen & Field, 1996; van Hateren & van der Schaaf, 1998). Interestingly, van Hateren and Ruderman (1998) found that Independent Components Analysis produced a range of spatial frequency tuning only when moving images were included in the training set. In the case of NBCM, we find that adjusting the parameters (*α* and *β*) affects the spatial frequency preferences of the neurons (Figs. 1–3); however, this affects all neurons together, rather than causing the neurons to better tile frequency space. It is possible that we could get more realistic tiling (as in V1) by adding more versatility to the NBCM mix. First, we could model the neurons so that they learn at different rates, say (equivalent to assigning different neurons different values of *α* and *β*). Second, we could model the LGN inputs to the learning fields as coming from both P- and M-cells, whose spatial frequency tuning differs in consequence of the 2–3 times difference in the diameters of their receptive-field centers (Croner & Kaplan, 1995; Derrington & Lennie, 1984). The separate P and M cell populations could project to different simple cells, or they could provide convergent input (Vidyasagar et al., 2002; Yoshioka, Levitt, & Lund, 1994).

We found that the orientation bandwidths of the NBCM neurons were a reasonable match for the V1 population. However, this result is somewhat dependent on the specific details of our NBCM simulation. When we also ran similar simulations using a 32 × 32-pixel grid (Fig. 7), some NBCM neurons then developed particularly elongated receptive fields with much lower orientation bandwidths than real neurons.

### 4.3 Comparison with other models of V1 receptive fields

Numerous other models have attempted to account for the structure of V1 receptive fields in terms of the statistical structure of visual input. Some of the most notable are the Gabor model (Daugman, 1980; Field & Tolhurst, 1986; Marcelja, 1980), the Olshausen and Field (1996), Olshausen and Field (1997) model, and ICA (Bell & Sejnowski, 1996; van Hateren & Ruderman, 1998; van Hateren & van der Schaaf, 1998). These models have succeeded to varying degrees in describing the parameters of V1 receptive fields. The Gabor model cannot be completely evaluated because it is too flexible – it models the general structure of V1 receptive fields but makes no prediction about their tuning preferences or bandwidths. Both the Olshausen-Field model and ICA capture the facts that V1 receptive fields have oriented bar-like structure, and have bandwidths that are roughly the same as those observed in cortex. A notable failure is that ICA RFs tend to have high spatial frequency tuning, whereas real V1 neurons show a range of preferences; this can be remedied by performing ICA on natural movies, rather than static images (van Hateren & Ruderman, 1998). The NBCM model performs similarly to these other models. However, it shows some quantization of orientation tuning (Fig. 3A–C) that is not observed in cortex. On the other hand, with an appropriate choice of parameters, NBCM RFs have more cortex-like bandwidths than ICA (Fig. 6).

### 4.4 What is contrast normalization for?

One view is that visual cortex develops neonatally so that neuronal receptive fields match the statistics of the visual input. When a young animal is presented with a limited range of visual inputs (Blakemore & Cooper, 1970; Blakemore & Van Sluyters, 1975), its neurons will learn to represent only this limited range; but when a wider range is presented, the neurons cooperate so that all the available stimulus energy is represented. The simulations presented here may model this process. We find that after approximately a million iterations, the NBCM rule produces stable receptive fields. If a real observer fixates one scene per second, this corresponds to approximately 275 h of viewing time – comparable to the 675 h of exposure (5 h per day for 4.5 months) found by Blakemore and Cooper to be effective in changing the orientation tuning of kitten cortical neurons.

However, the role of neonatal visual experience has been disputed (Stryker & Sherk, 1975). It is clear that animals have oriented V1 receptive fields from the moment that they first view the world (Carlson, Hubel, & Wiesel, 1986; Chapman, Godecke, & Bonhoeffer, 1999). Perhaps, neonatal viewing of an imbalanced range of orientations only disrupts or alters the orientation tuning of some subsets of V1 neurons but not of others (Leventhal & Hirsch, 1975; Li, Peterson, & Freeman, 2003). That ferret kits have orientation-tuned V1 neurons even before eye opening seems to imply that the scaffold, at least, of orientation tuning is inherent and is independent of visual experience. However, this tuning does rely on RGC activity (e.g. Chapman & Godecke, 2000) and it has been shown that the neurons of very young ferret kits actually respond to visual stimulation through their still-closed eyelids with some stimulus specificity (Krug, Akerman, & Thompson, 2001). For the early orientation tuning in V1, it is still not clear the relative contribution of early oriented visual stimulation though closed eyelids and of spontaneous RGC activity (driven by retinal waves – Wong, 1999).

The simulations presented here suggest that contrast normalization may play an important role in this process. On its own, the BCM rule provides a biologically plausible account of how individual neurons may learn to accurately represent visual input. It does not require an error signal to be fed back to the neurons to enable them to learn to accurately represent their input. Nor does it presume some preconception about the goal of coding or the nature of the image statistics that are key. The NBCM model extends this by adding a second biologically plausible element – contrast normalization (Heeger, 1992). The effect of contrast normalization is to discourage neurons from responding to identical structure in the visual input, and to disperse (distribute) coding across the neural population.

## Figures and Tables

**Table 1:** Coding properties of standard BCM and NBCM receptive fields.

	Standard BCM	NBCM (*α* = 1, *β* = 2)	NBCM (*α* = 2, *β* = 4)	ICA
Reconstruction error	0.61	0.31	0.44	0.28
Orthogonality	0.76	0.89	0.92	0.93
Rank	99 (/256)	175 (/256)	144 (/256)	168 (/256)
Lifetime sparseness	0.67	0.63	0.65	0.63
Population sparseness	0.42	0.44	0.48	0.52
Dispersal	0.66	0.64	0.64	0.37

## References

[b0005] Albrecht D.G., Hamilton D.B. (1982). Striate cortex of monkey and cat: Contrast response function. Journal of Neurophysiology.

[b0010] Bell A.J., Sejnowski T.J. (1997). The “independent components” of natural scenes are edge filters. Vision Research.

[b0015] Benardete E.A., Kaplan E. (1997). The receptive field of the primate P retinal ganglion cell, I: Linear dynamics. Visual Neuroscience.

[b0020] Bienenstock E.L., Cooper L.N., Munro P.W. (1982). Theory for the development of neuron selectivity: Orientation specificity and binocular interaction in visual cortex. Journal of Neuroscience.

[b0025] Blais B.S., Intrator N., Shouval H., Cooper L.N. (1998). Receptive field formation in natural scene environments: Comparison of single-cell learning rules. Neural Computation.

[b0030] Blakemore C., Cooper G.F. (1970). Development of the brain depends on the visual environment. Nature.

[b0035] Blakemore C., Van Sluyters R.C. (1975). Innate and environmental factors in the development of the kitten’s visual cortex. Journal of Physiology.

[b0040] Bonds A.B. (1989). Role of inhibition in the specification of orientation selectivity of cells in the cat striate cortex. Visual Neuroscience.

[b0045] Carandini M., Heeger D.J. (1994). Summation and division by neurons in primate visual cortex. Science.

[b0055] Carlson M., Hubel D.H., Wiesel T.N. (1986). Effects of monocular exposure to oriented lines on monkey striate cortex. Developmental Brain Research.

[b0050] Caywood M.S., Willmore B., Tolhurst D.J. (2004). Independent components of color natural scenes resemble V1 neurons in their spatial and color tuning. Journal of Neurophysiology.

[b0060] Chapman B., Godecke I. (2000). Cortical cell orientation selectivity fails to develop in the absence of on-center retinal ganglion cell activity. Journal of Neuroscience.

[b0065] Chapman B., Godecke I., Bonhoeffer T. (1999). Development of orientation preference in the mammalian visual cortex. Journal of Neurobiology.

[b0070] Cooper L.N., Intrator N., Blais B.S., Shouval H.Z. (2004). Theory of cortical plasticity.

[b0075] Croner L.J., Kaplan E. (1995). Receptive fields of P and M ganglion cells across the primate retina. Vision Research.

[b0080] Daugman J.G. (1980). Two-dimensional spectral analysis of cortical receptive field pro-files. Vision Research.

[b0085] De Valois R.L., Albrecht D.G., Thorell L.G. (1982). Spatial frequency selectivity of cells in macaque visual cortex. Vision Research.

[b0090] De Valois R.L., Yund E.W., Hepler N. (1982). The orientation and direction selectivity of cells in macaque visual cortex. Vision Research.

[b0095] DeAngelis G.C., Ohzawa I., Freeman R.D. (1993). Spatiotemporal organization of simple-cell receptive fields in the cat’s striate cortex. I. General characteristics and postnatal development. Journal of Neurophysiology.

[b0100] Derrington A.M., Lennie P. (1984). Spatial and temporal contrast sensitivities of neurones in lateral geniculate nucleus of macaque. Journal of Physiology.

[b0105] Falconbridge M.S., Stamps R.L., Badcock D.R. (2006). A simple Hebbian/anti-Hebbian network learns the sparse, independent components of natural images. Neural Computation.

[b0110] Field D.J. (1994). What is the goal of sensory coding?. Neural Computation.

[b0115] Field D.J., Tolhurst D.J. (1986). The structure and symmetry of simple-cell receptive-field profiles in the cat’s visual cortex. Proceedings of the Royal Society of London B.

[b0120] Fyfe C., Baddeley R. (1995). Finding compact and sparse-distributed representations in visual images. Network: Computation in Neural Systems.

[b0125] Geisler W.S., Albrecht D.G. (1992). Cortical neurons: Isolation of contrast gain control. Vision Research.

[b0130] Grossberg S. (1988). Nonlinear neural networks: Principles, mechanisms, and architectures. Neural Networks.

[b0135] Hancock P.J.B., Baddeley R.J., Smith L.S. (1992). The principal components of natural images. Network: Computation in Neural Systems.

[b0140] Heeger D.J. (1992). Normalization of cell responses in cat striate cortex. Visual Neuroscience.

[b0145] Hubel D.H., Wiesel T.N. (1959). Receptive fields of single neurones in the cat’s striate cortex. Journal of Physiology.

[b0150] Irvin G.E., Casagrande V.A., Norton T.T. (1993). Center/surround relationships of magnocellular, parvocellular, and koniocellular relay cells in primate lateral geniculate nucleus. Visual Neuroscience.

[b0155] Jones J.P., Palmer L.A. (1987). The two-dimensional spatial structure of simple receptive fields in cat striate cortex. Journal of Neurophysiology.

[b0160] Krug K., Akerman C.J., Thompson I.D. (2001). Responses of neurons in neonatal cortex and thalamus to patterned visual stimulation through the naturally closed lids. Journal of Neurophysiology.

[b0165] Law C.C., Cooper L.N. (1994). Formation of receptive fields in realistic visual environments according to the Bienenstock, Cooper, and Munro (BCM) theory. Proceedings of the National Academy of Sciences of the United States of America.

[b0170] Leventhal A.G., Hirsch H.V. (1975). Cortical effect of early selective exposure to diagonal lines. Science.

[b0175] Li B., Peterson M.R., Freeman R.D. (2003). The oblique effect: A neural basis in the visual cortex. Journal of Neurophysiology.

[b0180] Linsenmeier R.A., Frishman L.J., Jakiela H.G., Enroth-Cugell C. (1982). Receptive field properties of *x* and *y* cells in the cat retina derived from contrast sensitivity measurements. Vision Research.

[b0185] Marcelja S. (1980). Mathematical description of the responses of simple cortical cells. Journal of the Optical Society of America A.

[b0190] Marr D. (1982). Vision.

[b0195] Movshon J.A., Thompson I.D., Tolhurst D.J. (1978). Spatial and temporal contrast sensitivity of neurones in areas 17 and 18 of the cat’s visual cortex. Journal of Physiology.

[b0200] Movshon J.A., Thompson I.D., Tolhurst D.J. (1978). Spatial summation in the receptive fields of simple cells in the cat’s striate cortex. Journal of Physiology.

[b0205] Olshausen B.A., Field D.J. (1996). Emergence of simple-cell receptive field properties by learning a sparse code for natural images. Nature.

[b0210] Olshausen B.A., Field D.J. (1997). Sparse coding with an overcomplete basis set: A strategy employed by V1?. Vision Research.

[b0215] Schwartz O., Simoncelli E.P. (2001). Natural signal statistics and sensory gain control. Nature Neuroscience.

[b0220] Shouval H., Intrator N., Cooper L.N. (1997). BCM network develops orientation selectivity and ocular dominance in natural scene environment. Vision Research.

[b0225] Smyth D., Willmore B., Baker G.E., Thompson I.D., Tolhurst D.J. (2003). The receptive-field organization of simple cells in primary visual cortex of ferrets under natural scene stimulation. Journal of Neuroscience.

[b0230] Stryker M.P., Sherk H. (1975). Modification of cortical orientation selectivity in the cat by restricted visual experience: A reexamination. Science.

[b0235] Tolhurst D.J., Heeger D.J. (1997). Comparison of contrast-normalization and threshold models of the responses of simple cells in cat striate cortex. Visual Neuroscience.

[b0240] Tolhurst D.J., Smyth D., Thompson I.D. (2009). The sparseness of neuronal responses in ferret primary visual cortex. Journal of Neuroscience.

[b0245] Tolhurst D.J., Tadmor Y., Tang C. (1992). Amplitude spectra of natural images. Ophthalmology and Physiological Optics.

[b0250] Tolhurst D.J., Thompson I.D. (1981). On the variety of spatial frequency selectivities shown by neurons in area 17 of the cat. Proceedings of the Royal Society of London B.

[b0255] van Hateren J.H., Ruderman D.L. (1998). Independent component analysis of natural image sequences yields spatio-temporal filters similar to simple cells in primary visual cortex. Proceedings of the Royal Society of London B.

[b0260] van Hateren J.H., van der Schaaf A. (1998). Independent component filters of natural images compared with simple cells in primary visual cortex. Proceedings of the Royal Society of London B.

[b0265] Vidyasagar T.R., Kulikowski J.J., Lipnicki D.M., Dreher B. (2002). Convergence of parvocellular and magnocellular information channels in the primary visual cortex of the macaque. The European Journal of Neuroscience.

[b0270] Vinje W.E., Gallant J.L. (2000). Sparse coding and decorrelation in primary visual cortex during natural vision. Science.

[b0275] Wiesel T.N., Hubel D.H. (1963). Single-cell responses in striate cortex of kittens deprived of vision in one eye. Journal of Neurophysiology.

[b0280] Willmore B., Tolhurst D.J. (2001). Characterizing the sparseness of neural codes. Network: Computation in Neural Systems.

[b0285] Willmore B., Watters P.A., Tolhurst D.J. (2000). A comparison of natural-image-based models of simple-cell coding. Perception.

[b0290] Wong R.O. (1999). Retinal waves and visual system development. Annual Review of Neuroscience.

[b0295] Yoshioka T., Levitt J.B., Lund J.S. (1994). Independence and merger of thalamocortical channels within macaque monkey primary visual cortex: Anatomy of interlaminar projections. Visual Neuroscience.

